# IRF-5-Mediated Inflammation Limits CD8^+^ T Cell Expansion by Inducing HIF-1α and Impairing Dendritic Cell Functions during *Leishmania* Infection

**DOI:** 10.1371/journal.ppat.1004938

**Published:** 2015-06-05

**Authors:** Akil Hammami, Tania Charpentier, Mélina Smans, Simona Stäger

**Affiliations:** INRS—Institut Armand-Frappier, Laval, Quebec, Canada; Cornell University, UNITED STATES

## Abstract

Inflammation is known to be necessary for promoting, sustaining, and tuning CD8^+^ T cell responses. Following experimental *Leishmania donovani* infection, the inflammatory response is mainly induced by the transcription factor IRF-5. IRF-5 is responsible for the activation of several genes encoding key pro-inflammatory cytokines, such as IL-6 and TNF. Here, we investigate the role of IRF-5-mediated inflammation in regulating antigen-specific CD8^+^ T cell responses during *L*. *donovani* infection. Our data demonstrate that the inflammatory response induced by IRF-5 limits CD8^+^ T cell expansion and induces HIF-1α in dendritic cells. Ablation of HIF-1α in CD11c^+^ cells resulted into a higher frequency of short-lived effector cells (SLEC), enhanced CD8^+^ T cell expansion, and increased IL-12 expression by splenic DCs. Moreover, mice with a targeted depletion of HIF-1α in CD11c^+^ cells had a significantly lower splenic parasite burden, suggesting that induction of HIF-1α may represent an immune evasive mechanism adopted by *Leishmania* parasites to establish persistent infections.

## Author Summary

Inflammation is essential for inducing, sustaining, and regulating CD8^+^ T cell responses. The transcription factor IRF-5 is mainly responsible for initiating the inflammatory response following experimental *Leishmani donovani* infection. IRF-5 activates several genes encoding key pro-inflammatory cytokines, such as IL-6 and TNF. In this study, we investigate the role of IRF-5-mediated inflammation in regulating antigen-specific CD8^+^ T cell responses during *L*. *donovani* infection. Our data demonstrate that the inflammatory response induced by IRF-5 limits the expansion CD8^+^ T cell. This negative effect is mediated by the induction of HIF-1α in dendritic cells. Indeed, we observed a significant increase in CD8^+^ T cell expansion in mice lacking HIF-1α expression in dendritic cells. Moreover, these mice had a significantly lower parasite burden in the spleen, suggesting that induction of HIF-1α may represent an immune evasive mechanism adopted by *Leishmania* parasites to establish persistent infections.

## Introduction

Maintenance of a proper balance between inflammatory and anti-inflammatory responses is essential for achieving effective immunity against infectious pathogens while limiting collateral inflammatory damage to the tissue. However, immunosuppressive responses are sometimes generated in excess. This event often results in the strong inhibition of protective pro-inflammatory responses and leads to susceptibility to infectious pathogens, such as *Plasmodium* [1,2], *Leishmania* [3,4], lymphocytic choriomeningitis virus [5,6], and *Mycobacteria spp*. [7].

Visceral leishmaniasis (VL) is a good example of dysregulated balance between inflammatory and anti-inflammatory responses. VL is a potentially lethal disease caused by *Leishmania donovani* and *L*. *infantum/chagasi*. *Leishmania* are protozoan parasites, existing as flagellated promastigotes within sandflies and as intracellular amastigotes in infected mammals. In the host, *Leishmania* preferentially infects macrophages; however, it can also be found in other cells, such as DCs, neutrophils, and fibroblasts [8–12]. VL is characterized by persistent infection of the spleen and by immunodeficiency during the chronic stage [13]. Experimental infection with *L*. *donovani* results in pathogen-induced disruption of the splenic microarchitecture, which involves both the disruption of the marginal zone and the B-cell follicles, and the progressive loss of stromal cells [14,15]. This disruption is mediated by TNF [16], a cytokine that is overexpressed during VL [17,18]. Interestingly, TNF deficient mice infected with *L*. *donovani* have a lower IL-10 mRNA accumulation in the spleen than do their wild type counterparts [14], suggesting that TNF may be involved as a positive regulator of IL-10 production.

We have recently demonstrated that the inflammatory response following *L*. *donovani* infection is largely mediated by the transcription factor IRF-5 [19]. IRF-5 can be activated by TLR7 and TLR9 via the MyD88 signaling pathway and/or directly by viral infections and Type I interferon [20]. This transcription factor is responsible for the activation of genes encoding for various key inflammatory cytokines [21–24]. Interestingly, *L*. *donovani* infected *Irf5* ^*-/-*^ mice do not show the hallmark symptoms of VL, which are hepato-and splenomegaly, due to the lack of inflammatory cell infiltration. Furthermore, these mice generate profoundly defective Th1 responses during chronic disease [19]. The role of IRF-5-mediated inflammation on the development of CD8^+^ T cell responses during VL has not yet been explored.

We have previously shown that *L*. *donovani* induces defective CD8^+^ T cell responses with limited clonal expansion [25]. Moreover, the majority of CD8^+^ T cells that survive clonal contraction are central memory-like cells, suggesting that perhaps effector responses are not sustained.

Pro-inflammatory cytokines are known to tune CD8^+^ T cell responses and provide the critical third signal necessary for the development of effector CD8^+^ T cells [26–30]. For instance, IL-12 seems to regulate T-bet and eomesodermin (Eomes) expression [30,31], the differentiation of short-lived effector cells (SLEC) [30], and the cytolytic activity of CTLs [32]. Type I IFN, IFNγ, and IL-4 also appear to be required for efficient CD8^+^ T cells priming and memory differentiation [33–38]. The inflammatory milieu was also shown to control antigen sensitivity by enhancing T cell receptor signaling [39]. In contrast, Stelekati et al. recently reported that a bystander chronic inflammatory milieu impairs the development of CD8^+^ T cell memory following immunization [40]. This implies that inflammation does not always play a positive role in supporting the development of CD8^+^ T cell responses and that the role of inflammation might depend on the specific inflammatory milieu induced by each pathogen.

In this study, we investigated the role of IRF-5 mediated inflammation in regulating CD8^+^ T cell expansion following *L*. *donovani* infection. Our data shows that IRF-5 participates in limiting antigen-specific CD8^+^ T cell expansion at the very early stages of infection by indirectly inducing HIF-1α expression in DCs. Upregulation of HIF-1α in DCs resulted in decreased IL-12 and increased IL-10 expression. Ablation of HIF-1α in CD11c^+^ cells led to a higher frequency of short-lived effector CD8^+^ T cells (SLEC), enhanced CD8^+^ T cell expansion, and significantly reduced parasite burden.

## Results

### IRF-5-mediated inflammation limits CD8^+^ T cell expansion during acute infection

**Fig 1:**
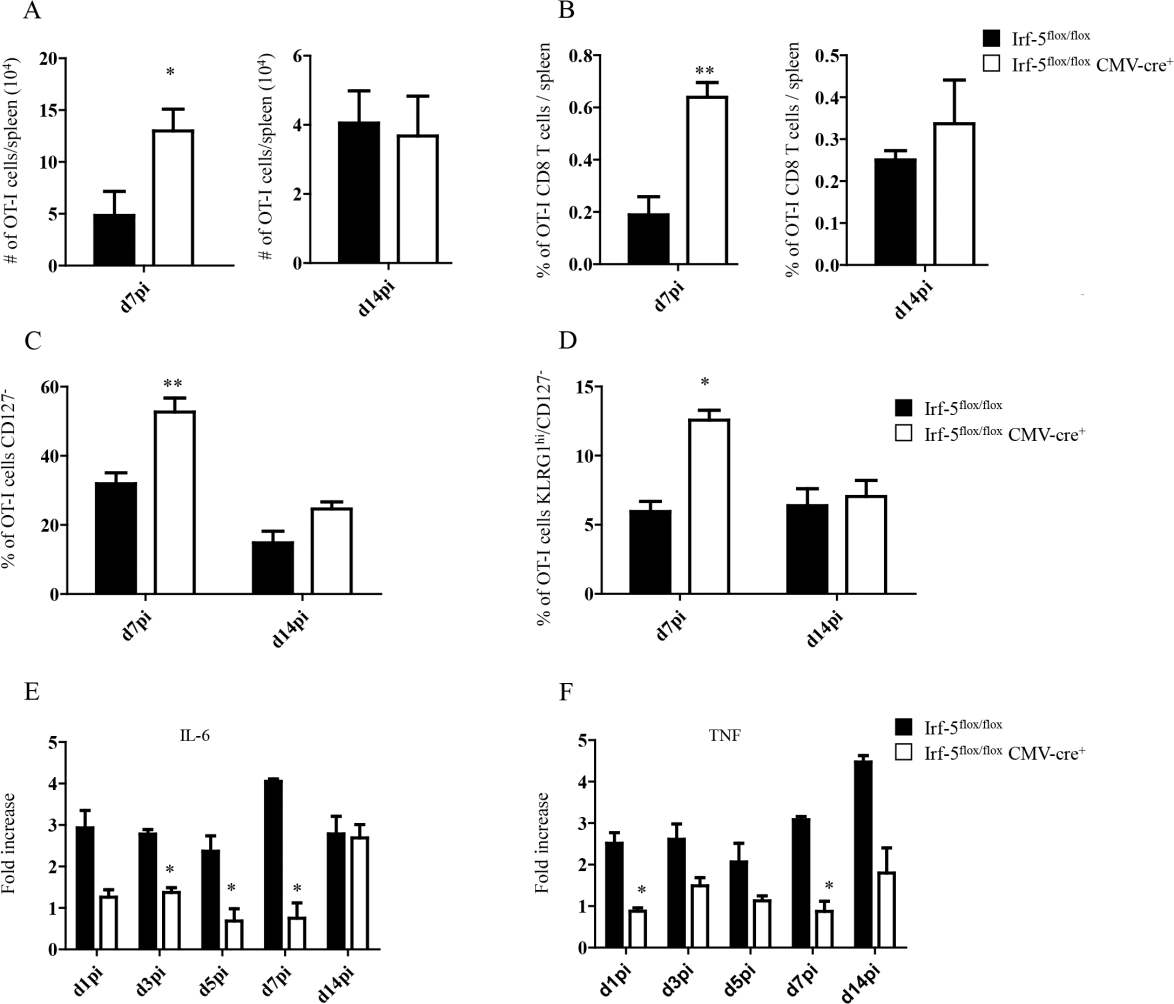
IRF-5-mediated inflammation limits CD8^+^ T cell expansion during acute *L*. *donovani* infection. (A-D) 2x10^**4**^ OT-I CD8^**+**^ T cells were adoptively transferred into recipient mice a day prior to infection with ovalbumin-transgenic (PINK) *L*. *donovani* amastigotes. (A) Graph represents the average number of OT-I CD8^**+**^ T cells found in the spleen from *Irf-5* ^***flox/flox***^ *CMV-Cre* ^***+***^ and *Cre* ^***-***^ mice at d7 and d14 p.i.. (B) Percentage of OT-I CD8^**+**^ T cells at d7 and d14 post infection. (C) Percentage of gated OT-I CD8^**+**^ that were negative for CD127. (D) Percentage of OT-I CD8^**+**^ T cells that did not express CD127 and are positive for KLRG1. Real-time PCR analysis of IL-6 (E) and TNF (F) expression in CD11c^**+**^ cells from *Irf-5* ^***flox/flox***^ *CMV-Cre* ^***+***^ and *Cre* ^***-***^ at various time points after infection. All data represent mean ± SEM of one of 3 independent experiments, n = 5. * denotes *p*<0.05, ** denotes *p*<0.01 and *** denotes p<0.001.

We have previously demonstrated that IRF-5 is essential for initiating the inflammatory response following experimental *L*. *donovani* infection. Indeed, *Irf5* ^*-/-*^ mice fail to generate mature granulomas in the liver and exhibit a severely reduced inflammatory infiltration in the liver and spleen [19]. Hence, to investigate the role of inflammation in the development of antigen-specific CD8^+^ T cells, we monitored CD8^+^ T cell responses in *L*. *donovani* infected IRF-5-deficient mice. To this end, we adoptively transferred CD45.1-OT-I CD8^+^ T cells into *Irf5* ^*flox/flox*^-*CMV-Cre* ^*-*^ and *Irf5* ^*flox/flox*^-*CMV-Cre* ^*+*^ mice. Mice were subsequently infected with ovalbumin-transgenic *L*. *donovani* amastigotes. We have previously reported that OT-I CD8^+^ T cells undergo clonal expansion between day 3 and 7 after parasite inoculation; by day 14, about 80% of the cells display a central memory-like phenotype [25]. To our surprise, we observed a 3–4 fold increase in the number (Fig 1A) and frequency (Fig 1B) of OT-I CD8^+^ T cells present in the spleen of IRF-5-deficient mice at d7p.i.; no difference was detected at d14p.i. We next analysed the phenotype of OT-I CD8^+^ T cells found in the spleen at d7 and 14p.i. Despite the fact that about 35% of OT-I CD8^+^ T cells had downregulated CD127 (Fig 1C and S1 Fig), only 7% of the OT-I cells were CD127^-^KLRG1^+^ in the *Cre* ^*-*^ group (Fig 1D and S1 Fig), suggesting that only a small percentage of the cells were short-lived effector cells (SLEC). In contrast, a significant increase in frequency of CD127^-^ (Fig 1C and S1 Fig) cells and SLEC (Fig 1D and S1 Fig) was noticed in IRF-5-deficient mice at d7 p.i.

Because *Leishmania* is known to induce strong IL-6 and TNF responses [17,18] and IRF-5 is known to govern the expression of both cytokines [21–24], we next assessed the mRNA levels for IL-6 and TNF in the spleen of *L*. *donovani* infected mice. As expected, IRF-5-deficient mice had a lower expression of IL-6 during the first week of infection (Fig 1E). By d14 p.i., however, both experimental groups expressed similar mRNA levels. In contrast, TNF mRNA levels were higher in *Cre* ^*-*^ mice compared to IRF-5 deficient mice during the first 2 weeks of infection (Fig 1F).

Taken together, our data suggest that IRF-5 participates in limiting CD8^+^ T cell expansion and hampering the development of SLEC.

### Upregulation of HIF-1α in the spleen restricts CD8^+^ T cell expansion

**Fig 2:**
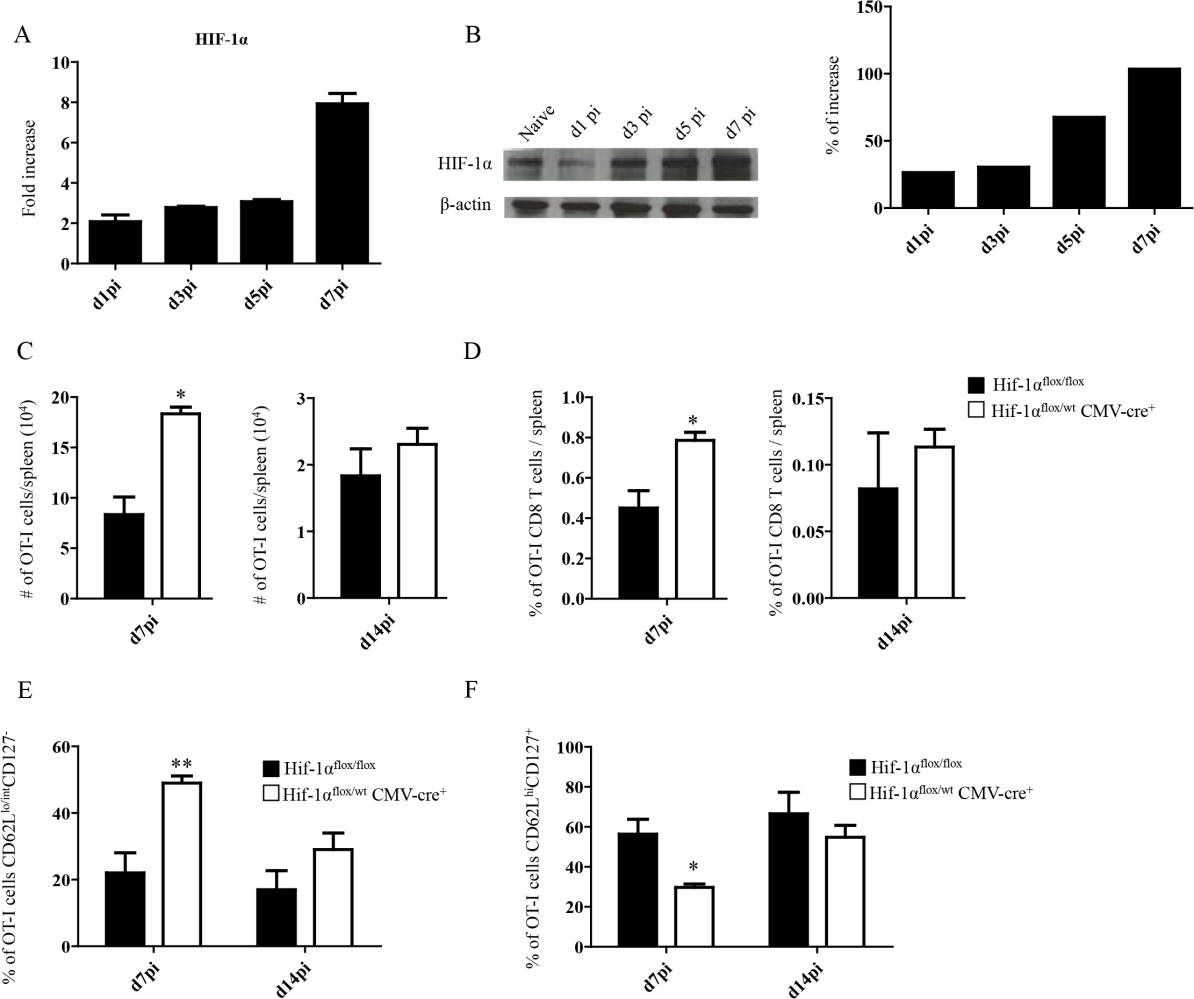
Upregulation of HIF-1α in the spleen restricts CD8^+^ T cell expansion. (A-B) Mice were infected with 2x10^**7**^ amastigotes intravenously and euthanized at various time points after infection. HIF-1α expression in splenocytes was assessed by real-time PCR (A) and immunoblot analysis (B). (C-F) 2x10^**4**^ OT-I CD8^**+**^ T cells were adoptively transferred into recipient mice a day prior to infection with ovalbumin-transgenic (PINK) *L*. *donovani* amastigotes. (C) Graphs represent the average number of OT-I CD8^**+**^ T cells found in the spleen from *Hif1a* ^***flox/flox***^ and *Hif1a* ^***+/-***^ *mice* at d7 and d14 p.i.. (D) Percentage of OT-I CD8^**+**^ T cells at d7 and d14 p.i.. (E) Percentage of gated OT-I CD8^**+**^ expressing CD62L^**lo/int**^ and negative for CD127. (F) Percentage of OT-I CD8^**+**^ T cells that were CD44^**+**^ CD127^**+**^CD62L^**hi**^. All data represent mean ± SEM of one of 3 independent experiments, n = 5. * denotes *p*<0.05 and ** denotes *p*<0.01.

Because inflammation is known to induce HIF-1α, we next proceeded to assess HIF-1α expression in splenocytes from *L*. *donovani* infected mice. The hypoxia-inducible transcription factor (HIF-1α) is the key regulator in the cellular response to hypoxia [41,42]. Under hypoxic conditions HIF-1α accumulates and translocates into the nucleus, where it binds the constitutively expressed HIF-1β [43]. The resultant heterodimer binds and triggers transcription of the hypoxia response element-containing genes in all cells [44]. Nevertheless, accumulation and transcriptional activity of HIF-1α can also be induced at normoxic conditions by pro-inflammatory cytokines such as TNF and IL-1β [45,46], or by TLR ligation [47–49]. Furthermore, *Leishmania* promastigotes were reported to induce HIF-1α in macrophages [50–52]. Hence, we first investigated whether HIF-1α expression was at all upregulated in splenocytes following the inoculation of *L*. *donovani* amastigotes. As expected, HIF-1α mRNA expression was upregulated in the spleen of *L*. *donovani* infected mice (Fig 2A). HIF-1α upregulation was confirmed on the protein level by western blot (Fig 2B). We next determined if the upregulation of HIF-1α in splenocytes had a negative effect on CD8^+^ T cell expansion and function. Thus, we generated hemizygous *Hif1a* ^*+/-*^ mice by crossing *Hif1a* ^*flox/flox*^ with *CMV-Cre* mice. We then monitored adoptively transferred CD45.1-OT-I CD8^+^ T cells at day 7 and 14 p.i. in *L*. *donovani* infected *Hif1a* ^*+/-*^ and *Hif1a* ^*flox/flox*^ mice. Interestingly, we observed a significant increase in the number (Fig 2C) as well as frequency (Fig 2D) of OT-I CD8^+^ T cells at day 7 p.i. in *Hif1a* ^*+/-*^ compared to the control group; at d14 p.i., however, both groups had similar number and frequency of OT-I CD8^+^ T cells in the spleen. As previously seen in IRF-5-deficient mice, increased expansion was paralleled by a higher frequency of effector cells (Fig 2E and S2 Fig). We also notice a significant decrease in the frequency of CD44^hi^CD62L^hi^CD127^+^ cells in *Hif1a* ^*+/-*^ mice compared to the control group (Fig 2F and S2 Fig). Collectively, our results suggest that HIF-1α might be involved in limiting CD8^+^ T cell expansion and effector cell differentiation.

### *L*. *donovani* infection induces HIF-1α expression in CD11c^hi^ splenic DCs in an IRF-5-dependent manner

**Fig 3:**
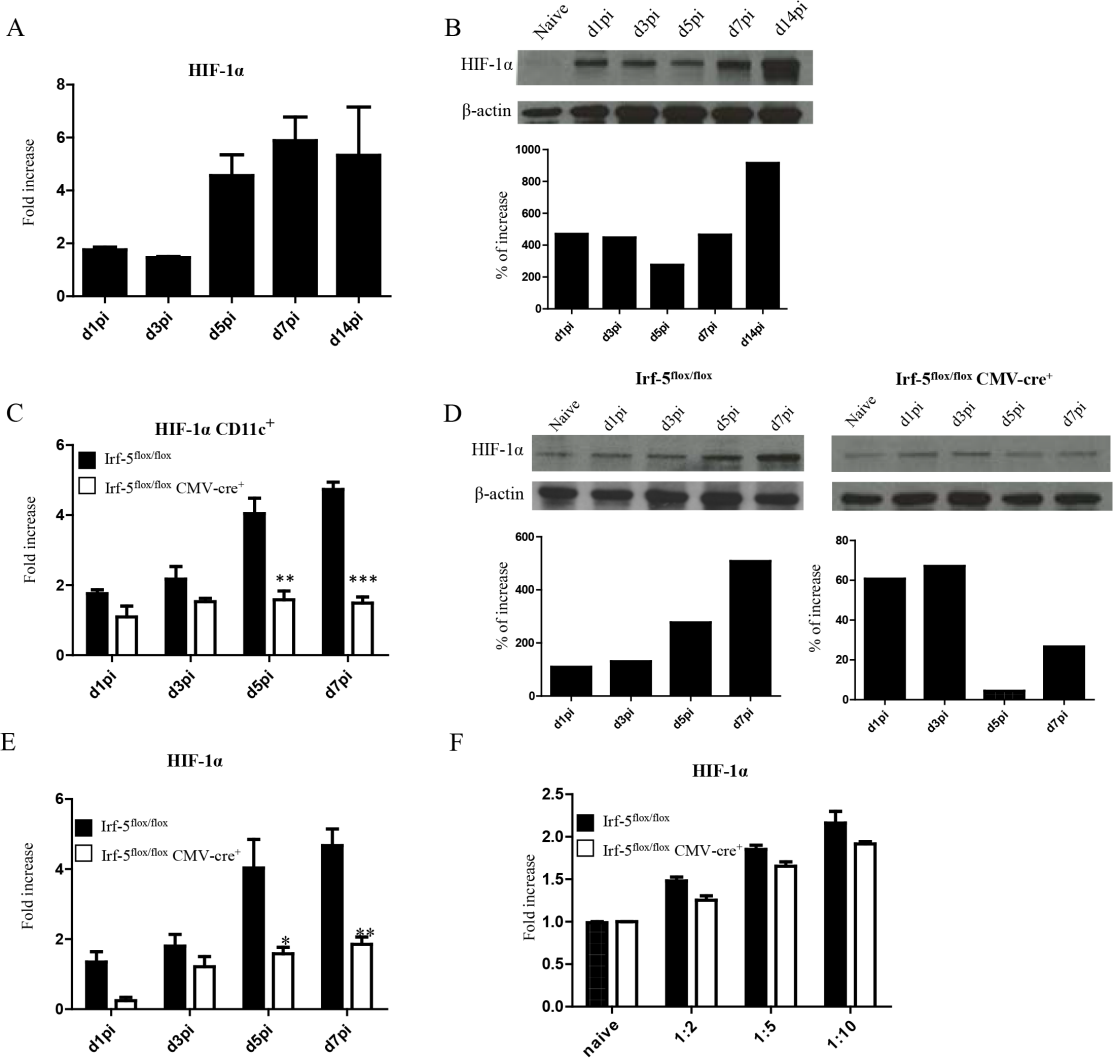
*L*. *donovani* infection induces HIF-1α expression in CD11c^hi^ splenic DCs in an IRF-5 dependent manner. Mice were infected with 2x10^**7**^ amastigotes intravenously. (A) Real-time PCR analysis of HIF-1α mRNA expression in CD11c^**+**^ cells purified from C57BL/6 mice at various time points after infection. (B) Immunoblot analysis of HIF-1α expression in CD11c^**+**^ cells from C57BL/6 mice (upper panel) and densitometric analysis normalized to ß-actin expression and expressed as fold increase to results obtained with naïve mice (lower panel). (C) Real-time PCR analysis of HIF-1α expression in sorted CD11c^**+**^ cells from *Irf-5* ^***flox/flox***^ *Cre* ^***-***^ and *Irf-5* ^***flox/flox***^ *CMV-Cre* ^***+***^. (D) Immunoblot analysis of *Hif-1α* expression in CD11c^**+**^ cells population of *Irf5* ^***flox/flox***^ *Cre* ^***-***^ (left upper panel) and *Irf-5* ^***flox/flox***^ *CMV-Cre* ^***+***^ (right upper panel), and densitometric analysis normalized to ß-actin expression and expressed as fold increase to results obtained with naïve mice (lower panels). (E) Real-time PCR analysis of Hif-1α expression in CD11c^**-**^ splenocytes from *Irf-5* ^***flox/flox***^ *Cre* ^***-***^ and *Irf-5* ^***flox/flox***^ *CMV-Cre* ^***+***^. (F) Real-time PCR analysis of HIF-1α mRNA expression in BMDC from *Irf-5* ^***flox/flox***^ *CMV-Cre* ^**+**^ and *Cre* ^**-**^ mice. All data represent mean ± SEM combined from 3 independent experiments.

Enhanced CD8^+^ T cell responses were only observed at peak expansion in *Hif1a* ^*+/-*^ mice. Hence, it is possible that this transcription factor exerts different functions among various cell types. Since dendritic cells play a crucial role during T cell priming, we next assessed the effect of HIF-1α expression in these cells. First, we wanted to determine if HIF-1α was at all upregulated in dendritic cells during *L*. *donovani* infection. As shown in Fig 3A, HIF-1α was progressively expressed in DCs during the first week of infection. Increased expression was also confirmed on the protein level by western blot (Fig 3B). To determine whether IRF-5-mediated inflammation was at all involved in HIF-1α upregulation in DCs, we next assessed HIF-1α mRNA levels in DCs from IRF-5 deficient mice following *L*. *donovani* infection. Interestingly, HIF-1α expression was only upregulated in DCs purified from infected IRF-5-sufficent mice, but not in those purified from IRF-5 deficient mice (Fig 3C and 3D), suggesting that IRF-5 is involved in the induction of HIF-1α in DCs. Similar results were obtained when HIF-1α expression was assessed in CD11c^-^ splenocytes (Fig 3E). HIF-1α was not directly induced by IRF-5 in DCs, since we could observe an increase in mRNA levels for HIF-1α in IRF-5 deficient bone marrow derived DCs infected with *L*. *donovani in vitro* (Fig 3F). This implies that IRF-5 is not required for the induction of HIF-1α when DCs are directly infected by the parasite. Because only a very small percentage of splenic DCs bury parasites during acute infection (S3 Fig), accumulation of HIF-1α mRNA in DCs is most likely due to the inflammatory milieu induced by IRF5.

### HIF-1α expression in CD11c^+^ cells limits expansion of CD8^+^ T cells and favours the induction of MPEC

HIF-1α upregulation in DCs was shown to reduce their stimulatory capacity for T cell functions in vitro [53]. To investigate the role of HIF-1 α expression in DCs during experimental VL, we generated HIF-1α conditional knock-out mice by crossing *Hif1a* ^*flox/flox*^ with *Cd11c-Cre* mice. Firstly, we assessed whether HIF-1α expression was abolished in our conditional knock-outs. As shown in S4A Fig, HIF-1α mRNA levels did not increase in *L*. *donovani* infected BMDC from *Hif1a* ^*flox/flox*^—*Cd11c-Cre* ^*+*^ mice compared to the *Cre* ^*-*^ group as measured by real time PCR. Similar results were obtained with CD11c^+^ cells purified from the spleen following in vivo infection with *L*. *donovani* (S4B Fig).

**Fig 4:**
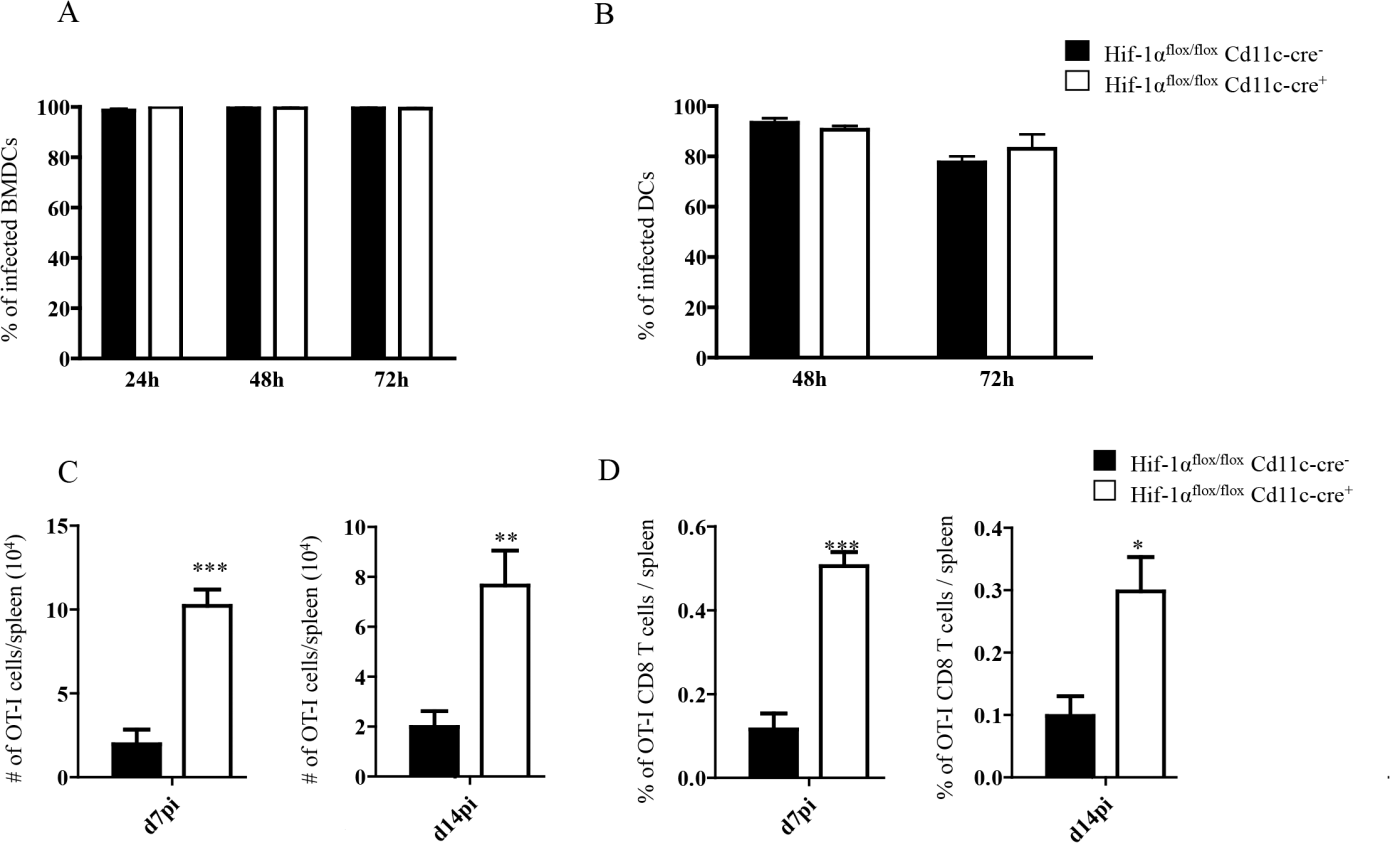
HIF-1α expression in CD11c^+^ cells limits expansion of CD8^+^ T cells. (A-B) Percentage of *in vitro* infected *Hif-1α* ^***flox/flox***^ *Cd11c-Cre* ^***-***^ and *Hif-1α* ^***flox/flox***^ *Cd11c-Cre* ^***+***^ BMDC (A) and CD11c^**+**^ cells (B). (C) 2x10^**4**^ OT-I CD8^**+**^ T cells were adoptively transferred into recipient mice a day prior to infection with 2x10^**7**^ovalbumin-transgenic (PINK) *L*. *donovani* amastigotes. Graph represents the average number of OT-I CD8^**+**^ T cells found in the spleen from *Hif-1α* ^***flox/flox***^ *Cd11c-Cre* ^***+***^ and *Cre* ^***-***^ at d7 p.i. (left panel) and d14 p.i. (right panel). (D) Percentage of OT-I CD8^**+**^ T cells at d7 p.i. (left panel) and d14 p.i. (right panel). All data is presented as the mean ± SEM of one of three independent experiments, n = 5. * denotes *p*<0.05, ** denotes *p*<0.01 and *** denotes p<0.001.

It has been reported that *Leishmania* promastigotes require HIF-1α for their survival inside macrophages and DCs [50,51]. Thus, we next evaluated whether this transcription factor was also needed for survival inside DCs by the intracellular and clinically relevant form of the parasite, namely the amastigote. In contrast to promastigotes, amastigotes seemed to survive equally well in BMDC from *Hif1a* ^*flox/flox*^—*Cd11c-Cre* ^+^ mice compared to those derived from the *Cre* ^*-*^ group (Fig 4A). We also assessed amastigote survival in purified splenic CD11c^hi^ DCs and found no differences at any time point between the two groups of mice (Fig 4B). This suggests that amastigotes were able to survive inside DCs from *Hif1a* ^*flox/flox*^—*Cd11c-Cre* ^*+*^ mice.

**Fig 5:**
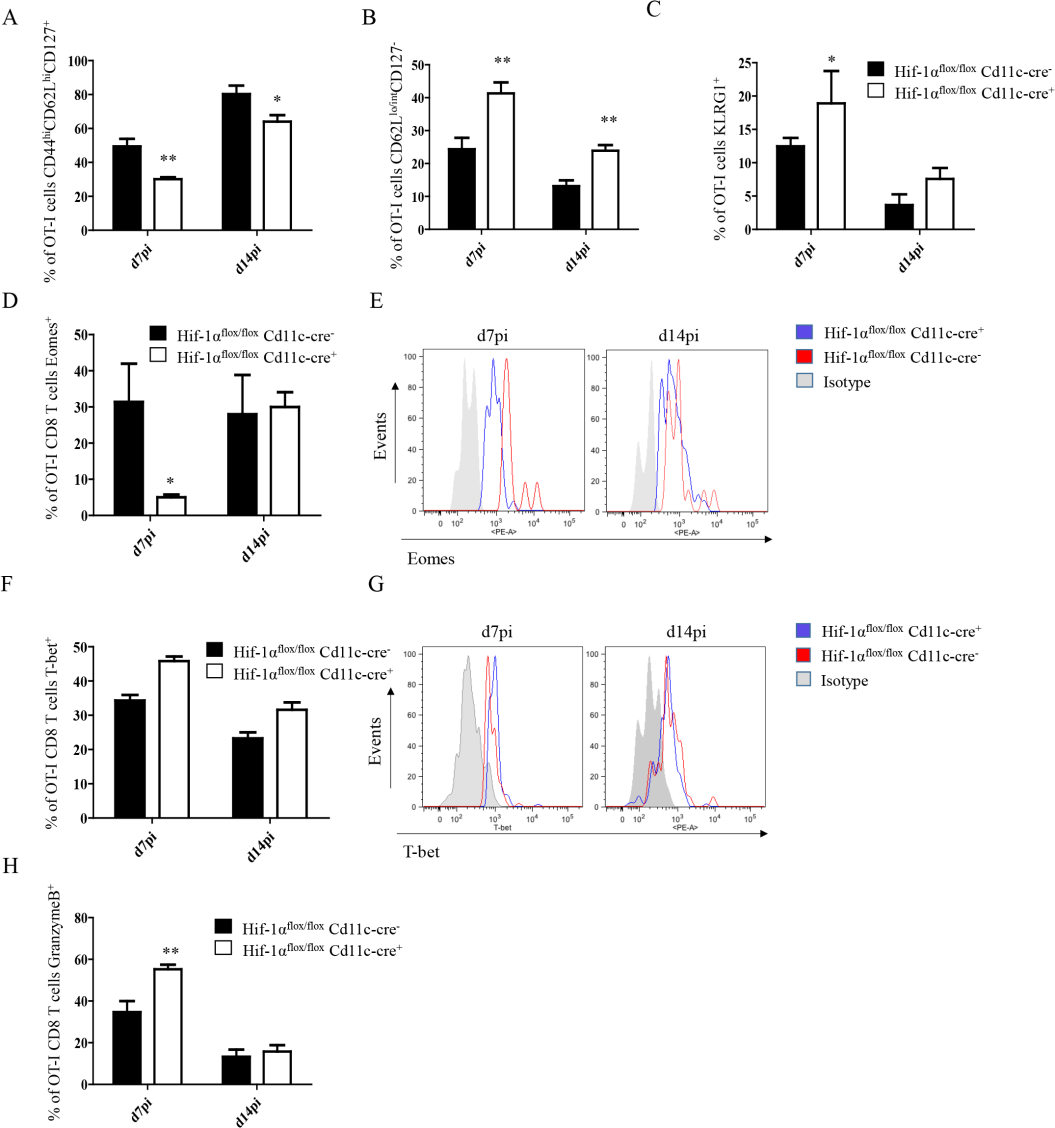
Depletion of HIF1α in CD11c^+^ cells induces more SLECs during the acute phase of *L*. *donovani* infection. 2x10^**4**^ OT-I CD8^**+**^ T cells were adoptively transferred into *Hif-1α* ^***flox/flox***^ *Cd11c-Cre* ^***-***^ and *Hif-1α* ^***flox/flox***^ *Cd11c-Cre* ^***+***^ mice. One day after, mice were infected with ovalbumin-transgenic (PINK) *L*. *donovani* amastigotes. OT-I CD8^**+**^ T cells were identified by gating on CD8^**+**^ CD45.1 cells from spleen of infected mice at d7 and 14 p.i. (A) Graph represents the frequency of CD44^**hi**^ CD62L^**hi**^ CD127^**+**^ OT-I CD8^**+**^ T cells. (B) Percentage of gated OT-I CD8^**+**^ cells that expressed low/intermediate levels of CD62L and were negative for CD127. (C) Percentage of OT-I CD8^**+**^ T cells positive for KLRG1. (D and F) Percentage of OT-I CD8^**+**^ T cells positive for Eomes (D) and T-bet (F). (E and G) Representative histograms for Eomes (E) and T-bet (G) staining for OT-I CD8^**+**^ T cells at day 7 and 14 p.i. (H) Percentage of OT-I CD8^**+**^ T cells positive for granzyme B. All data is presented as the mean ± SEM of one of three independent experiments, n = 5. * denotes *p*<0.05, ** denotes *p*<0.01 and *** denotes p<0.001.

We next proceeded to assess CD8^+^ T cell responses in our conditional knock-out mice. CD45.1-OT-I CD8^+^ T cells were adoptively transferred into *Hif1a* ^*flox/flox*^—*Cd11c-Cre* ^*+*^ mice and their *Cre* ^*-*^ littermates a day prior to infection with *L*. *donovani* amastigotes. CD8^+^ T cell responses were then monitored at d7 (peak expansion) and 14 p.i. (end of contraction). Remarkably, OT-I CD8^+^ T cells underwent a greater expansion in *Hif1a* ^*flox/flox*^—*Cd11c-Cre* ^*+*^ mice compared to their *Cre* ^*-*^ littermates as indicated by a larger number and higher frequency of cells present in the spleen at d7 p.i. (Fig 4C and 4D, left panels). Unlike in IRF-5 deficient and *Hif* ^*+/-*^ mice, this difference was still observed at d14 p.i., when OT-I CD8^+^ T cell numbers were 3–4 fold higher in the conditional knock-out group (Fig 4C and 4D, right panels). When we analyzed the phenotype of the adoptively transferred cells, we noticed a significant decrease in CD44^hi^CD62L^hi^CD127^+^ OT-I CD8^+^ T cells in *Hif1a* ^*flox/flox*^—*Cd11c-Cre* ^*+*^ mice compared to littermate controls at both time points analyzed (Fig 5A and S5 Fig), suggesting that less memory precursor effector cells (MPEC) were generated in those mice. These results were paralleled by an increase in effector cell frequency at d7 and 14 p.i. (Fig 5B and S5 Fig), which was reflected in a higher percentage of CD127^-^KLRG1^+^ cells (Fig 5C and S5 Fig) in *Hif1a* ^*flox/flox*^—*Cd11c-Cre* ^*+*^.

The T-box transcription factors Eomes and T-bet appear to regulate effector/memory differentiation program of CD8^+^ T cells. Because we observed an increase in frequency of SLEC, we were wondering whether Eomes and T-bet were expressed at different level in OT-I CD8^+^ T cells adoptively transferred into *Hif1a* ^*flox/flox*^—*Cd11c-Cre* ^*+*^ mice compared to their *Cre* ^*-*^ counterpart. As expected, we noticed a significant decrease in Eomes in *Hif1a* ^*flox/flox*^—*Cd11c-Cre* ^*+*^ mice at d7 p.i. compared to the HIF-1α sufficient group (Fig 5D and 5E). No differences, however, were observed at d14. A light upregulation of T-bet was also observed in OT-I CD8^+^ T cells from the *Cre* ^*+*^ group compared to the *Cre* ^*-*^ littermates, but this was not statistically significant (Fig 5F and 5G).

Interestingly, OT-I CD8^+^ T cells also showed a higher cytotoxic capacity in *Hif1a* ^*flox/flox*^—*Cd11c-Cre* ^*+*^ mice compared to the control group (Fig 5H and S5C Fig), while maintaining similar levels of IFNγ, TNF, and IL-2 production upon restimulation in vitro. Taken together, our data suggest that HIF-1α expression in CD11c^+^ cells favours the development of MPEC and limits CD8^+^ T cell expansion following *L*. *donovani* infection.

### HIF-1α hampers IL-12 expression by splenic CD11c^hi^ DCs

**Fig 6:**
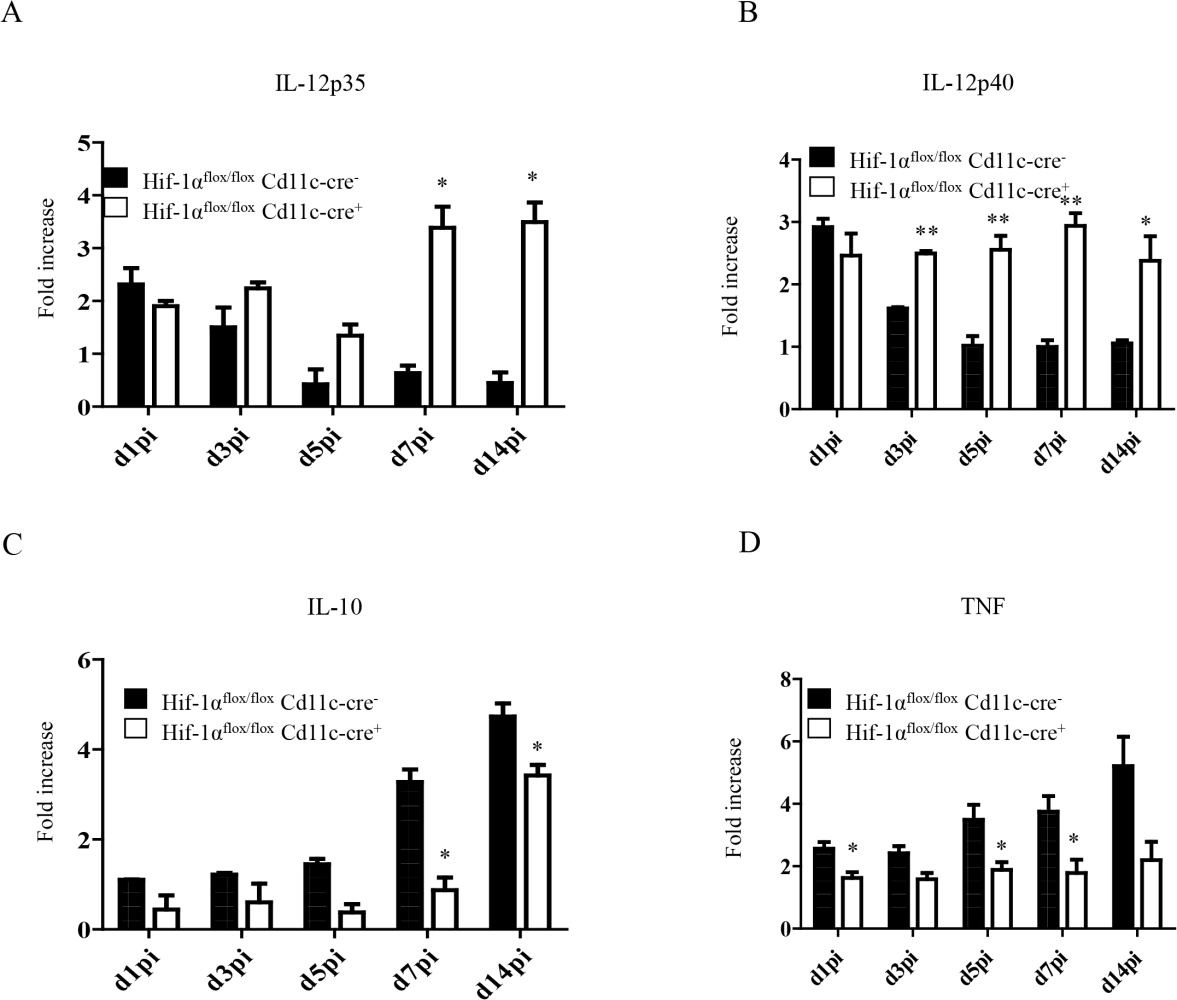
HIF-1α hampers dendritic cell function. Mice were infected with 2x10^**7**^ amastigotes intravenously. Real-time PCR analysis of IL-12p35 (A), IL-12p40 (B), IL-10 (C), and TNF (D) expression in CD11c^**+**^ cells from *Hif-1α* ^***flox/flox***^ *Cd11c-Cre* ^***-***^ and *Hif-1α* ^***flox/flox***^ *Cd11c-Cre* ^***+***^ mice over the course of infection. All data represent mean ± SEM combined from 3 independent experiments.

IL-12 plays an important role in promoting CD8^+^ T cell responses. Particularly, this cytokine has been shown to induce the development of SLEC by regulating T-bet expression [30,31]. In VL, IL-12 is mainly produced by DCs, however this production is not sustained after 24h [17]. Because more SLEC with a greater cytotoxic capacity were observed in *Hif1a* ^*flox/flox*^—*Cd11c-Cre* ^*+*^ mice, we were wondering whether HIF-1α deficient DCs were producing more IL-12. Hence, we purified CD11c^hi^ splenic DCs from *L*. *donovani* infected mice at various time points after infection and assessed the expression of IL-12p35 by real time PCR. As shown in Fig 6A, IL-12p35 expression was sustained in *Hif1a* ^*flox/flox*^—*Cd11c-Cre* ^*+*^ mice compared to the *Cre* ^*-*^ control group. Similar results were obtained when we measured IL-12p40 mRNA levels (Fig 6B).

We also assessed the expression of IL-10, a cytokine that is known to be associated with susceptibility to experimental VL. IL-10 mRNA accumulation is readily detected in DCs few days after *L*. *donovani* inoculation and is continuously produced until the chronic phase of the disease [54]. In agreement with the literature, we noticed an increase in IL-10 mRNA levels in the *Cre* ^*-*^ group during the first 2 weeks of infection (Fig 6C). In contrast, DCs from *Hif1a* ^*flox/flox*^—*Cd11c-Cre* ^*+*^ mice failed to upregulate IL-10 expression during the first week of infection. At d14 p.i., however, IL-10 mRNA levels in the *Cre* ^*-*^ group increased but were still significantly lower than the control group. No differences were observed between both groups in the IL-10 mRNA levels of the CD11c negative fraction. Interestingly, CD11c^hi^ splenic DCs lacking HIF-1α also expressed lower TNF mRNA levels compared to their HIF-1α-sufficient counterparts (Fig 6D); similarly to IL-10, TNF mRNA levels of the CD11c negative fraction were comparable in both groups between d5 and 14 p.i. (S6 Fig).

### HIF-1α expression in CD11c^+^ cells exacerbates disease

**Fig 7:**
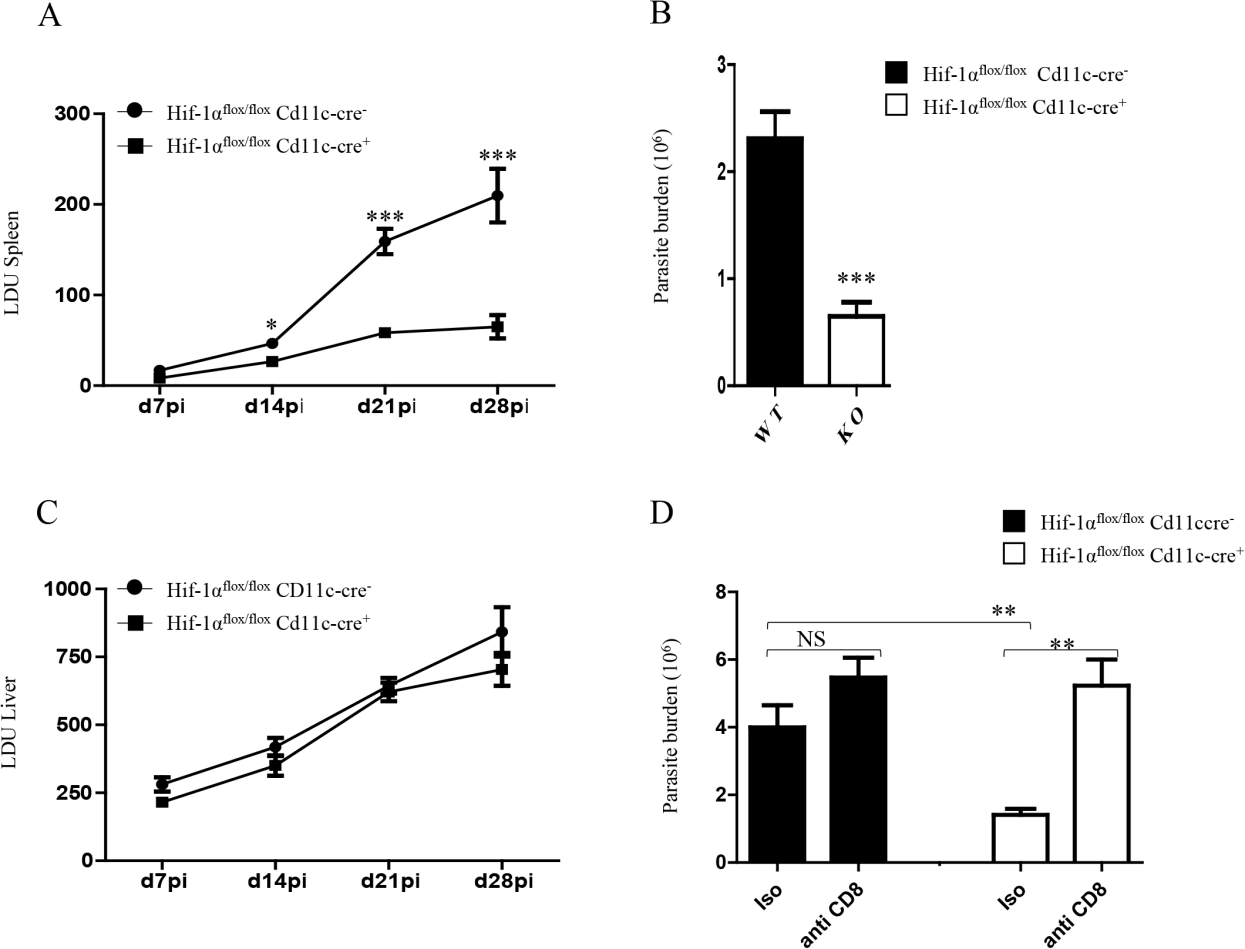
HIF-1α expression in CD11c^+^ cells exacerbates disease. *Hif-1α* ^***flox/flox***^ *Cd11c-Cre* ^***-***^ and *Hif-1α* ^***flox/flox***^ *Cd11c-Cre* ^***+***^ mice were infected i.v. with 2x10^**7**^ PINK *L*. *donovani* amastigotes. (A) Graph represents the splenic parasite burden expressed as Leishman Donovan Units (LDU) at various time points after infection. (B) The splenic parasite burden was determined by limiting dilutions at d 14 p.i. (C) Graph represents the hepatic parasite burden at different times of infection. (D) Graph shows the splenic parasite burden at d14 p.i. in infected *Hif-1α* ^***flox/flox***^ *Cd11c-Cre* ^***-***^ and *Hif-1α* ^***flox/flox***^ *Cd11c-Cre* ^***+***^ mice; mice were treated or not with the anti-CD8 antibody clone 2.43. All data is presented as the mean ± SEM, n = 5. * denotes *p*<0.05, ** denotes *p*<0.01 and *** denotes p<0.001.

Finally, we wanted to determine whether HIF-1α ablation in DCs had any biological effect on the course of *L*. *donovani* infection. Hence, we assessed the splenic parasite burden at various time points after infection (Fig 7A). We noticed a significant reduction in the splenic parasite burden in *Hif1a* ^*flox/flox*^—*Cd11c-Cre* ^*+*^ mice compared to the *Cre* ^*-*^ littermates already at d14p.i. Reduction in the parasite number in *Hif1a* ^*flox/flox*^—*Cd11c-Cre* ^*+*^ mice at d14p.i was also confirmed by limiting dilutions (Fig 7B). Interestingly, the hepatic parasite burden in *Hif1a* ^*flox/flox*^—*Cd11c-Cre* ^*+*^ mice was not different than their *Cre-* counterpart (Fig 7C). Finally, we proceeded to determine whether CD8^+^ T cells participate at all to the reduction in parasite numbers observed in the HIF-1α conditional knock-out group. Hence, we depleted CD8^+^ T cells in *L*. *donovani* infected *Hif1a* ^*flox/flox*^—*Cd11c-Cre* ^*+*^ and *Cre* ^*-*^ mice, and assessed the parasite burden at d14 p.i. As shown in Fig 7D, CD8^+^ T cell depletion only caused a mild and not significant increase in the parasite burden in Cre- mice; in contrast, CD8^+^ T cells seem to play an essential role in controlling parasite growth in HIF-1α conditional knockout mice, suggesting that HIF-1α induction in DCs is responsible for limiting protective CD8^+^ T cell responses.

In conclusion, HIF-1α induction in DCs represents an immune-evasive mechanism adopted by *Leishmania* to establish chronic infection.

## Discussion

The natural reaction of an organism to a foreign pathogen is to initiate an inflammatory response. This response is essential for effective immunity against the pathogen. In the present study though, we show that the IRF-5-dependent inflammatory milieu induced by *Leishmania* during the first week of infection inhibits CD8^+^ T cell expansion and the development of SLECs by inducing HIF-1α in dendritic cells and consequently altering DC functions. Ablation of HIF-1α in CD11c^+^ cells resulted in a lower parasite burden.

Following infection, antigen-specific CD8^+^ T cells expand and acquire effector function necessary for protective immunity. This process is initiated and sustained by inflammatory cytokines which play a major role during the development of CD8^+^ T cell responses by providing “signal 3”. Indeed, inflammatory cytokines were reported to tune effector/memory differentiation, expansion, and survival of antigen-specific CD8^+^ T cells [26–30,55]. IL-12, for instance, is crucial for effector function acquisition following infection with *Listeria monocytogenes* [55], vaccinia virus [56], and *Toxoplasma gondii* [57]. IL-12 was also shown to participate in the induction of T-bet while repressing Eomes [30,31,58]. In contrast, IL-12 deficiency does not seem to interfere with CD8^+^ T cell development following LCMV infection, where type I IFN play the most critical role [36,37]. Hence, it appears that inflammatory cytokines promote and maintain CD8^+^ T cell differentiation to effectors while preventing the development of memory cells. It is important to note that most of the work on the role of inflammation in promoting CD8^+^ T cells was done in models of acute infection. A recent work showed that a chronic inflammatory environment negatively impacted the development of CD8^+^ T cell responses [40], suggesting that perhaps the composition of the inflammatory milieu rather than inflammation itself determines the outcome of CD8^+^ T cell responses. It is thus crucial to identify the pathogen-specific signature of proinflammatory cytokines to determine the effect of inflammation on CD8^+^ T cell responses. Our data suggest that IRF-5-mediate inflammation induced by *Leishmania* at early stages of infection plays a detrimental role in the development of protective CD8^+^ T cell responses.

IRF-5 is involved in the transcriptional activation of both Type I IFN genes and genes encoding for key pro-inflammatory cytokines such as IL-12, TNF, IL-23 and IL-6 [21,22,24,59]. Our recent study using *Irf5* ^*-/-*^ mice indicates that IRF-5 is essential for the development of protective Th1 responses. In fact, *L*. *donovani* infected *Irf5* ^*-/-*^ mice generate profoundly defective Th1 cells during chronic disease. However, we observed stronger Th1 responses in *Irf5* ^*-/-*^ mice during the first two weeks of infection, suggesting that priming of Th1 cells was more effective in the absence of IRF-5 and that IRF-5 is mainly needed for sustaining Th1 responses [19]. These observations prompted us to think that the strong TNF-dominated inflammatory response induced by *Leishmania* at the onset of infection [18] [60] plays a dual role and may help the establishment of chronic infection by inhibiting T cell responses.

To control the inflammatory response launched by foreign pathogens upon encounter with the immune system, the host has evolved several defense mechanisms aimed at tissue protection. One of these mechanisms is the hypoxia-driven, adenosine receptor-mediated immune suppressive pathway. The hypoxia-adenosinergic tissue-protecting pathway is a physiologic response to inflammatory damage, low oxygen tension, and hypoxia-driven accumulation of extracellular adenosine. HIF-1α is the key regulator in the cellular response to hypoxia [41]. This pathway is designed to allow the cells to survive in an environment of low oxygen tension, but it also leads to suppression of pro-inflammatory responses [61–64]. Indeed, HIF-1α suppresses cytokine production in T-cells [64–66], induces the potency and the number of CD25^+^CD4^+^ regulatory T-cells [67], induces IL-10 production [68,69], and inhibits DC maturation [53]. Moreover, HIF-1α expression in tumor-associated macrophages enhances the T cell suppressive capacity of these cells [70,71]; HIF-1α also causes upregulation of PD-L1 on MDSC, exacerbating their suppressive capacity [72,73].

Under normoxic conditions, HIF-1α can be upregulated by inflammatory cytokines such as TNF and IL-1β, by TLR agonists, and by several viruses [74,75], bacteria [75,76], and parasites [51,75,77,78]. *Leishmania* promastigotes are known to stabilize HIF-1α and induce HIF-1α mRNA in host cells [51,52]. Our results indicate that *L*. *donovani* amastigotes are also able to induce HIF-1α mRNA in BMDC; however, unlike for promastigotes [51], HIF-1α was not required for amastigote survival inside DCs. Interestingly, HIF-1α mRNA was induced in dendritic cells following *L*. *donovani* infection in an IRF-5-dependent manner. Because HIF-1α mRNA levels in IRF-5 deficient BMDC infected with *L*. *donovani* amastigotes were comparable to those observed in infected IRF-5 sufficient BMDC, we assume that HIF-1α in DCs is mainly induced by the inflammatory milieu generated by IRF-5 rather than the parasite itself. This suggests that IRF-5 is not involved in the induction of HIF-1α in DCs upon direct infection with *L*. *donovani*. The factor(s) responsible for the induction of HIF-1α in DCs following infection with *L*. *donovani* amastigotes are yet unknown. We tried to block IL-6R and TNF, but this blockade did not affect HIF-1α expression or CD8^+^ T cell expansion, suggesting that the upregulation of HIF-1α in DCs does not rely on the effect of a single cytokine. Further investigations are warranted in order to identify the mechanism(s) of HIF-1α induction in DCs during experimental VL.

HIF-1α appears to play a dual role in myeloid cells. An important body of literature has demonstrated that HIF-1α is involved in myeloid cell-mediated inflammation and is critical in the defence against various pathogens [76,79,80]. For instance, macrophages phagocytose and kill bacteria better under hypoxic conditions rather than under normoxic conditions [76,80]. Thus it is not surprising that mice with a targeted deletion of HIF-1α in myeloid cells are more susceptible to many bacterial and viral pathogens [76,79,81]. In contrast, some pathogens such as *Toxoplasma gondii* and *Leishmania* (promastigotes) require HIF-1α for their survival inside the cell [50–52,78]. This transcription factor was also shown to enhance replication of some viruses [82–84]. In the tumor microenvironment, HIF-1α expression in tumor-associated macrophages suppresses T cell responses [70] and induces expression of arginase 1 [71]; HIF-1α also enhances the inhibitory effect of MDSC [72].

Divergent effects of HIF-1α upregulation have also been observed in DCs. HIF-1α appears to play an important role in DC differentiation and migration in a hypoxic environment [53,85]. Nevertheless, this transcription factor impairs the upregulation of CCR7, a chemokine involved in homing of mature DCs to secondary lymphoid organs [53,85]. Moreover, HIF-1α acts as a negative regulator of plasmacytoid dendritic cell development [86]. Our results reveal a detrimental role for HIF-1α in DC function during experimental VL. Indeed, the absence of HIF-1α in DCs resulted in increased CD8 T cell expansion and a higher frequency of SLECs. Increased expansion directly correlated with a lower splenic parasite burden in HIF-1α conditional knock out mice. In contrast, the parasite load in the liver remained unchanged. This is possibly due to the differences in the cellular environment between liver and spleen, and also to the type of cell that hosts the parasite at the onset of infection. The two organs generate very diverse immune responses that lead to parasite clearance in the liver and chronic infection in the spleen [13].

Interestingly, OT-I CD8^+^ T cells were still detectable at a higher frequency at d14 p.i. in *Hif* ^*flox/flox*^- *Cd11c-cre* ^+^ mice; in contrast in *Hif* ^*+/-*^mice, the frequency at d14 p.i. was similar to the control group. This difference could possibly be caused by the general reduction in the level of IL-10 expression in infected *Hif* ^*+/-*^ mice compared to *Hif* ^*flox/flox*^- *Cd11c-cre* ^+^ mice. We have previously reported that IL-10 blockade induces increased expansion of CD8^+^ T cells [87]. However, in the absence of IL-10, CD8^+^ T cells display a more dramatic contraction, at the end of which CD8^+^ T cell numbers are similar to those observed in infected wild type mice. This suggests that HIF-1α expression in some cells is necessary to counterbalance the strong inflammatory responses induced by *L*. *donovani* and slow down clonal contraction.

*L*. *donovani* typically induces a swift IL-12 production by DCs at very early stages of infection; this production, however, ceases after 24h of infection [17]. We observed a sustained IL-12p35 and IL12p40 expression in HIF-1α deficient DCs. This expression was paralleled by a decrease in IL-10 and TNF mRNA levels. This suggests that HIF-1α directly or indirectly regulates the expression of IL-12, TNF, and IL-10 in dendritic cells. Increased expression of IL-12p35 and p40 may explain the higher frequency of SLECs detected in mice with a targeted deletion of HIF-1α in CD11c^+^ cells and therefore the enhanced expansion. Identifying the mechanism of HIF-1α induction in DCs will help to understand the physiological role of this transcription factor. It is possible that the pathway of activation as well as the duration of the signal determine the target gene selection and the final effect on DC functions.

In conclusion, we demonstrate that IRF-5-mediated inflammation induced by *L*. *donovani* at the onset of the infection participates in limiting CD8^+^ T cell expansion by upregulating HIF-1α in DCs and subsequently impairing DC functions. Several pathogens that cause chronic infections induce upregulation of HIF-1α, which is then directly required for their survival. Our data additionally shows that HIF-1α induction in DCs that are not the main target of *Leishmania* results in disease susceptibility. It is thus tempting to speculate that targeting the HIF-1α pathway may represent a major immune evasive mechanism adopted by some pathogens to establish chronic infection.

## Materials and Methods

### Mice and parasites

C57BL/6-*Tg(OT-I)-RAG1* ^*tm1Mom*^ mice and B6-Ly5.1 congenic mice were purchased from The Jackson Laboratory. Mice hemizygous for HIF-1α were generated by crossing once *Hif1a* ^flox/flox^ mice with mice expressing the cre-recombinase under the CMV promoter. Mice with a targeted HIF-1α mutation in CD11c^+^ cells were generated by crossing C57BL/6 *Hif1a* ^flox/flox^ mice with mice expressing the cre-recombinase under the CD11c promoter (C57BL/6 *Cd11c-Cre* ^*+/-*^), both purchased by The Jackson Laboratory. IRF-5-deficient mice were generated by crossing C57BL/6 *Irf5* ^*flox/flox*^ mice with mice expressing the cre-recombinase under the CMV promoter (The Jackson Laboratory). C57BL/6 *Irf5* ^*flox/flox*^ mice were a kind gift from Dr. Paula Pitha-Rowe (The Johns Hopkins University). All mice were housed at the INRS animal facility under specific pathogen-free conditions and used at 6–10 weeks of age. Ovalbumin-transgenic parasites were a gift from Drs. P. Kaye and D.F. Smith (University of York, UK). Wild type and ovalbumin transgenic *Leishmania donovani* (strain LV9) were maintained by serial passage in B6.129S7-*Rag1* ^*tm1Mom*^ mice, and amastigotes were isolated from the spleens of infected animals. Mice were infected by injecting 2×10^7^ amastigotes intravenously via the lateral tail vein. Splenic parasite burdens were determined either by limiting dilutions or by examining methanol-fixed, Giemsa stained tissue impression smears [87]. Data are presented as number of parasites per spleen or as Leishman Donovan Units (LDU).

### Ethics statement

Experiments involving mice were carried out under protocols approved by the Comité Institutionel de Protection des Animaux of the INRS-Institut Armand-Frappier (1110–06, 1110–07). These protocols respect procedures on good animal practice provided by the Canadian Council on animal care.

### Adoptive transfer of OT-I cells

CD45.1-OT-I/RAG1 mice, transgenic for a T cell receptor specific for chicken ovalbumin 257–264 presented by the MHC class I molecule H-2 K^b^, were used as T cell donors. CD8^+^ T cells were enriched from splenocytes of naïve CD45.1-OT-I/RAG1 animals as previously described [25]. 2x10^4^ CD45.1- OT-I CD8^+^ T cells were injected into the lateral tail vein of *Irf5* ^*flox/flox*^ CMV-*Cre* ^*+*^, *Hif1a* ^*+/-*^, *Hif1α* ^*flox/flox*^ *CD11c-Cre* ^*+*^ mice and their respective *Cre* ^*-*^ littermate controls. Animals were infected the day after with ovalbumin-transgenic *Leishmania donovani* amastigotes.

### PKH67 labelling of parasites

*L*. *donovani* parasites were stained with PKH67 (Sigma) following manufacturer’s instructions, as previously described [87]. Mice received 5 x 10^7^ PKH67 labeled parasites. Spleens from naïve and infected mice were harvested 24h later and surface stained for flow cytometric analysis.

### Flow cytometry

Adoptively transferred OT-I CD8^+^ T cells were identified by staining splenocytes with FITC-conjugated anti-CD45.1 antibody and Pacific Blue-conjugated anti-CD8 (BD Biosciences). The following antibodies were used to further characterize the OT-I response: APC-conjugated anti-CD44 and anti-KLRG1, PE-Cy5-conjugated anti-CD62L, and PE-conjugated anti-CD127 (all obtained from eBioscience). For all surface markers, cells were directly stained as previously described [25]. For intracellular staining, splenocytes were stimulated with the SIINFEKL peptide for 4 hours in the presence of Brefeldin A and recombinant IL-2, and then stained with FITC-conjugated anti-CD45.1 and Pacific Blue-conjugated anti-CD8. After fixation, cells were permeabilized and stained with PE-conjugated anti-granzyme B (Invitrogen), PE-conjugated anti-Eomes and anti-IL-2 (BD Biosciences). APC-conjugated anti-INFγ and PE-Cy7-conjugated anti-TNF were also used. Flow cytometric analysis was performed with a *BD* LSRFortessa cell analyzer (Becton Dickinson). One to two millions cells per sample were acquired and analyzed with the FACSDiva or with the Flowjo software.

For DCs analysis after injection of PKH67-labelled parasites, spleens were removed 24h after parasite injection and digested with collagenase. Splenocytes were then stained with anti-CD11c-APC, anti-MHCII-PE, anti-CD8-PB, and anti-CD4-PE-Cy7. Flow cytometric analysis was performed with a *BD* LSRFortessa cell analyzer (Becton Dickinson).

### Real-time PCR analysis

Real-time PCR (Stratagene mx3005p Real time PCR System) was used to analyze transcripts levels of HPRT, HIF-1α, IL-10, IL-12p35, IL-12p40, IL-6, and TNF [19,54,88,89]. Total RNA was insolated using RNeasy (Qiagen) to perform real-time RT-PCR. cDNA was prepared using 500 ng of total RNA using High capacity cDNA Reverse Transcription kit (Bio Rad). Real time PCR was performed using standard cycle of amplification.

### Western blot analysis

Total cell protein extracts of CD11c^+^ cells purified by MACS and cell sorting from infected and naive mice were pooled and lysed in RIPA buffer (sigma Aldrich, Germany). Equal amounts of protein (15 μg) were fractionated by 10% SDS-PAGE. Monoclonal anti-HIF-1α antibody Hif-1α67 (Novus Biologicals, Littleton, CO, USA) was used for immunoblot assays. Blots were stripped and reprobed with a polyclonal antibody against β-actin to confirm equal protein loading. [90]**.** Densitometric analysis was performed by spot densitometry using AlphaImager 3400 imaging software (Alpha Innotech Corporation) and normalized to ß-actin control. Values are presented as fold induction compared to the level in naive mice.

### Survival of *L*. *donovani* following *in vitro* infection

CD11c^+^ dendritic cells (DCs) were isolated using anti-CD11c beads (Miltenyi Biotec) [19]. BMDC were generated as previously described [91] and seeded at 10^6^ cells/ml onto 24-well plates. Cells were subsequently infected with PKH26-stained *L*. *donovani* amastigotes (Sigma, staining done according to manufacturer’s instructions) at a MOI of 5:1. Infected cells were then harvested 24, 48, and 72 h later, fixed with 2% PFA, stained with Hoechst (Invitrogen, staining done according to manufacturer’s instructions) and cytospined on PBS/ 1% BSA-prepared slides. Mounted slides were than analyzed using a fluorescent microscope, to evaluate parasite survival and target infection rate.

### CD8 T cell depletion

*Hif* ^*flox/flox*^- *Cd11c* cre^+^ and cre^-^ mice were treated bi-weekly with 0.2 mg of anti-CD8 antibody (clone 2.43), starting one day before infection. At day 14 p.i., mice were sacrificed and parasite burdens were determined by limiting dilutions.

### Statistical analysis

Statistical analysis was performed using a Student’s t-test, with p<0.05 considered significant. All experiments were conducted independently at least three times.

## Supporting Information

S1 Fig (A) Representative FACS plots depicting the gating strategy used to detect adoptively transferred CD45.1^+^ CD8^+^ OT-I cells. (B) Modulation of expression of CD127 at d7 (upper panels) and 14 p.i. (lower panels). Representative FACS plot for *Irf5* ^*flox/flox*^ *CMV-Cre* ^*+*^(left panels) and *Irf5* ^*flox/flox*^ *CMV-Cre* ^*-*^ (right panels) (C) Modulation of expression of CD127 and KLRG1 at d7 (upper panels) and 14 p.i. (lower panels). Representative FACS plot for *Irf5* ^*flox/flox*^ *CMV-Cre* ^*+*^ (left panels) and *Irf5* ^*flox/flox*^ *CMV-Cre* ^*-*^ mice (right panels).
(TIF)

S2 Fig (A) Modulation of expression of CD127 and CD62L at d7 (upper panels) and 14 p.i. (lower panels). Representative FACS plot for *Hif-1α* ^*flox/WT*^ *CMV-Cre* ^*+*^ (left panels) and *Hif-1α* ^*flox/flox*^ mice (right panels).
(TIF)

S3 Fig Mice were infected with 5x10^7^ PKH67-labelled *L*. *donovani* amastigotes intravenously and sacrificed 24h later. The percentage of PKH67^+^ DCs was determined by flow cytometry.
(TIF)

S4 Fig (A) Real-time PCR analysis of HIF-1α mRNA expression in *Hif-1α* ^*flox/*^ *floxCd11c-Cre* ^*-*^ and *Hif-1α* ^*flox/flox*^ *Cd11c-Cre* ^*+*^ BMDC infected *in vitro* at a MOI of 1:2, 1:5, and 1:10. (B) Real-time PCR analysis of HIF-1α mRNA expression in sorted CD11c^+^ cells from *Hif-1α* ^*flox/flox*^ *Cd11c-Cre* ^*-*^ and *Hif-1α* ^*floxlfox*^ *Cd11c-Cre* ^*+*^ mice over the course of infection.
(TIF)

S5 Fig (A) Modulation of expression of CD127 and CD62L at d7 (upper panels) and 14 p.i. (lower panels). Representative FACS plot for *Hif-1α* ^*flox/flox*^ *Cd11c-Cre* ^*+*^ (left panels) and *Hif-1α* ^*flox/flox*^ *Cd11c-Cre* ^*-*^ mice (right panels). (B) Modulation of expression of KLRG1 at d7 (upper panels) and 14 p.i. (lower panels). Representative FACS plot for *Hif-1α* ^*flox/flox*^ *Cd11c-Cre* ^*+*^ (left panels) and *Hif-1α* ^*flox/flox*^ *Cd11c-Cre* ^*-*^ mice (right panels). (C) Representative FACS plots for granzyme B expression at d7 (upper panels) and d14 p.i. (lower panels) in *Hif-1α* ^*flox/flox*^ *Cd11c-Cre* ^*+*^ (left panels) and *Hif-1α* ^*flox/flox*^ *Cd11c-Cre* ^*-*^ mice (right panels).
(TIF)

S6 Fig Mice were infected with 2x10^7^ amastigotes intravenously. Real-time PCR analysis of I TNF expression in CD11c^-^ cells from *Hif-1α* ^*flox/flox*^ *Cd11c-Cre* ^*-*^ and *Hif-1α* ^*flox/flox*^ *Cd11c-Cre* ^*+*^ mice over the course of infection. All data represent mean ± SEM combined from 3 independent experiments.
(TIF)
