# Supplementary figures and images for: IRF-5-Mediated Inflammation Limits CD8+ T Cell Expansion by Inducing HIF-1α and Impairing Dendritic Cell Functions during Leishmania Infection
Source: PLoS Pathog. 2015 Jun 5;11(6):e1004938. doi: 10.1371/journal.ppat.1004938 (PMC4457842; doi:10.1371/journal.ppat.1004938)

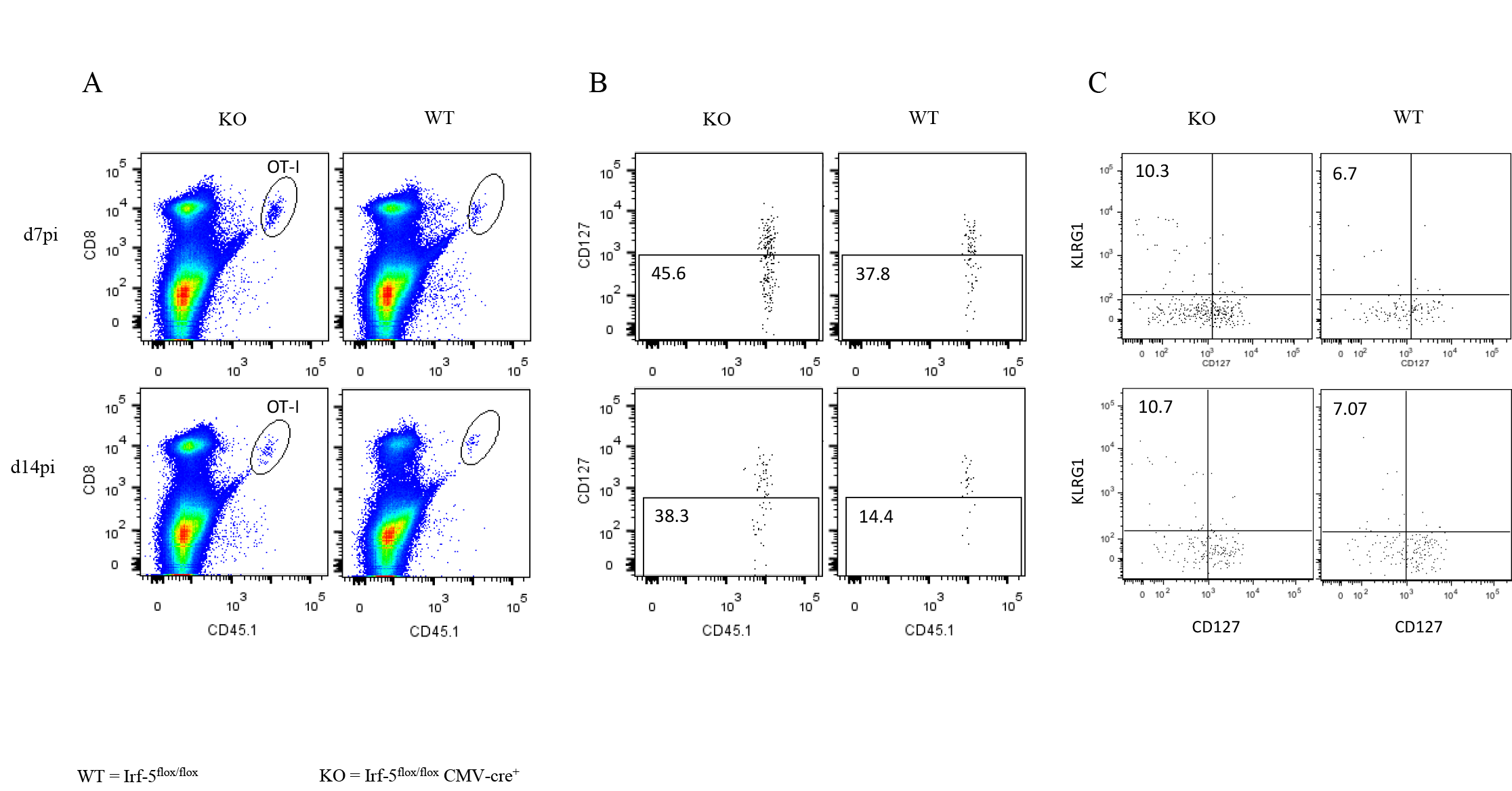

Supplement: S1 Fig — (B) Modulation of expression of CD127 at d7 (upper panels) and 14 p.i. (lower panels). Representative FACS plot for Irf5 flox/flox CMV-Cre +(left panels) and Irf5 flox/flox CMV-Cre - (right panels) (C) Modulation of expression of CD127 and KLRG1 at d7 (upper panels) and 14 p.i. (lower panels). Representative FACS plot for Irf5 flox/flox CMV-Cre + (left panels) and Irf5 flox/flox CMV-Cre - mice (right panels). (TIF) [file ppat.1004938.s001.tif]

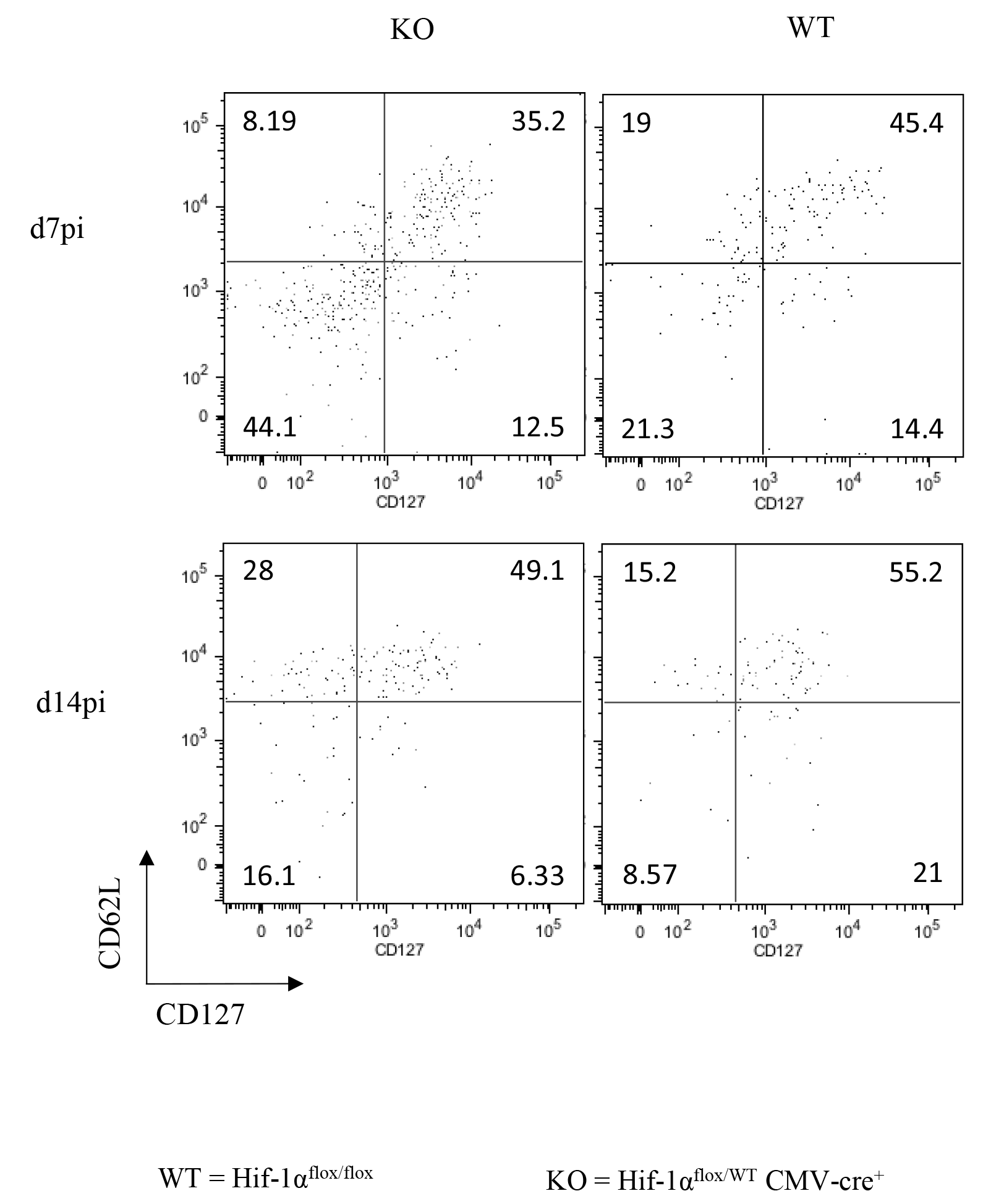

Supplement: S2 Fig — Representative FACS plot for Hif-1α flox/WT CMV-Cre + (left panels) and Hif-1α flox/flox mice (right panels). (TIF) [file ppat.1004938.s002.tif]

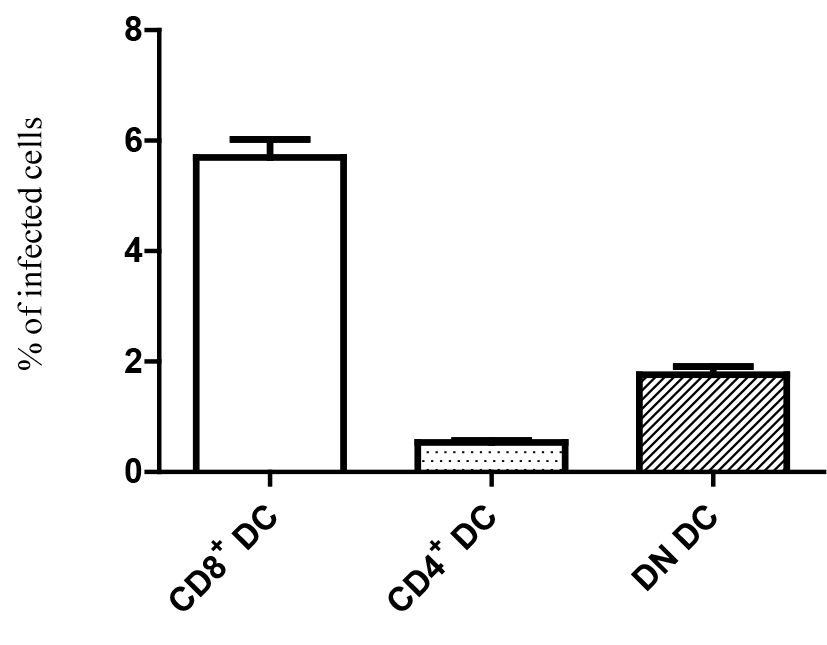

Supplement: S3 Fig — The percentage of PKH67+ DCs was determined by flow cytometry. (TIF) [file ppat.1004938.s003.tif]

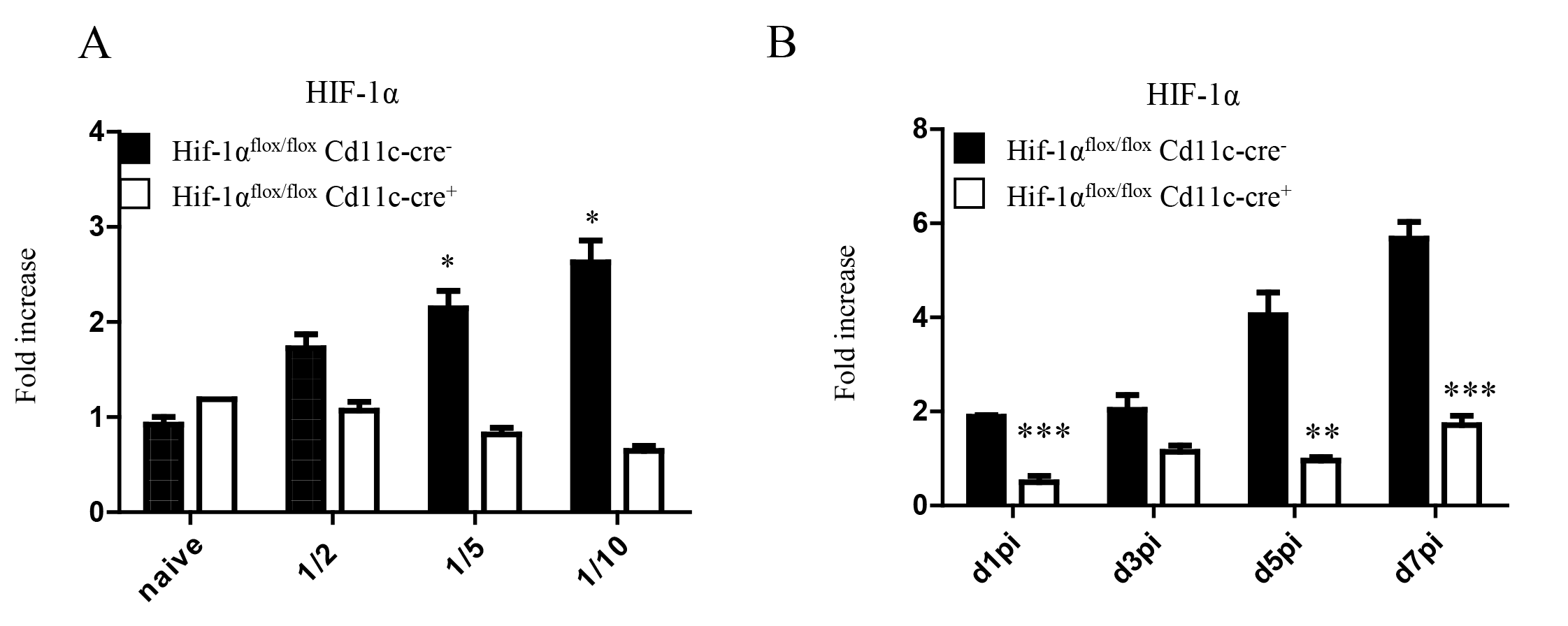

Supplement: S4 Fig — (B) Real-time PCR analysis of HIF-1α mRNA expression in sorted CD11c+ cells from Hif-1α flox/flox Cd11c-Cre - and Hif-1α floxlfox Cd11c-Cre + mice over the course of infection. (TIF) [file ppat.1004938.s004.tif]

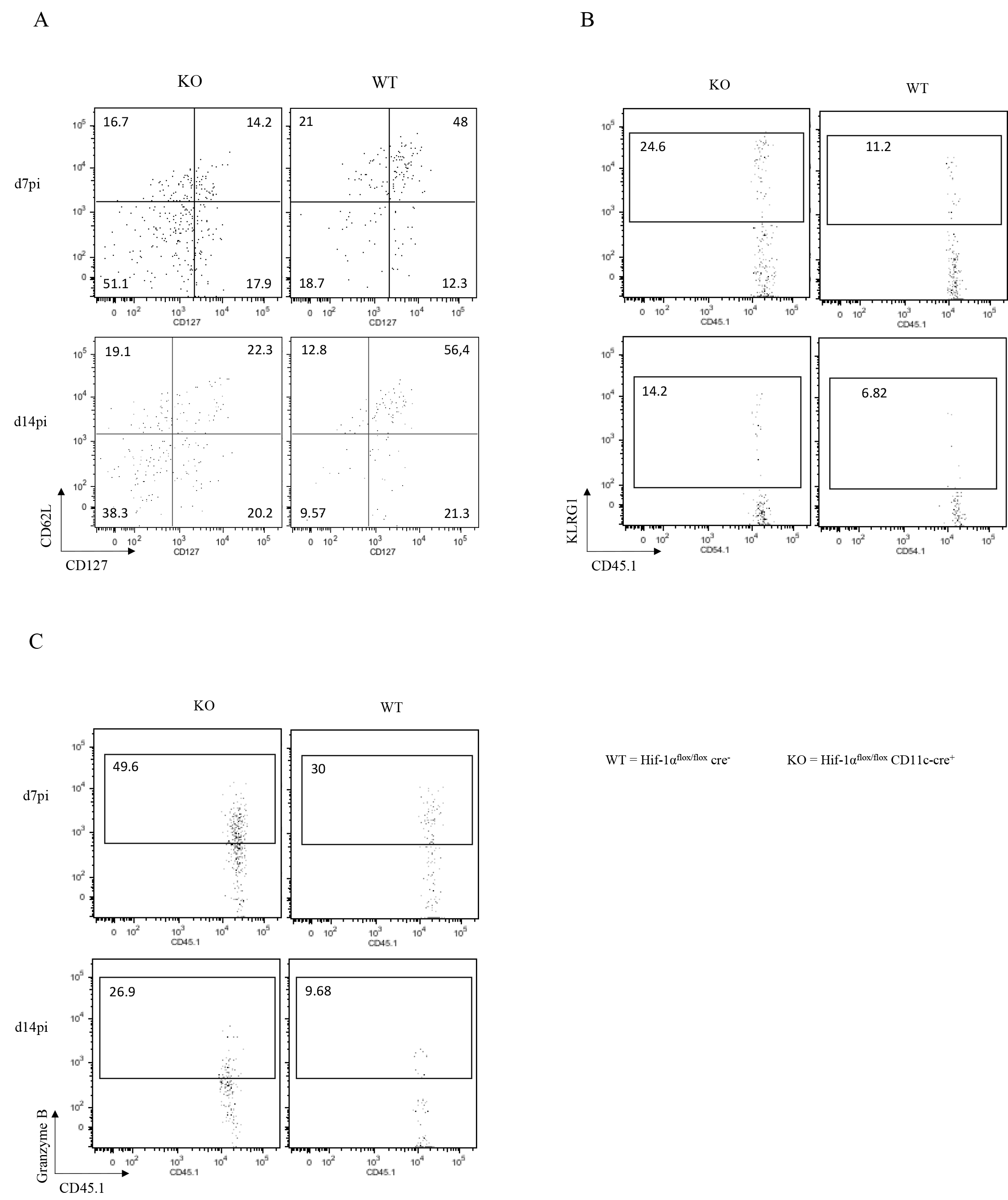

Supplement: S5 Fig — Representative FACS plot for Hif-1α flox/flox Cd11c-Cre + (left panels) and Hif-1α flox/flox Cd11c-Cre - mice (right panels). (B) Modulation of expression of KLRG1 at d7 (upper panels) and 14 p.i. (lower panels). Representative FACS plot for Hif-1α flox/flox Cd11c-Cre + (left panels) and Hif-1α flox/flox Cd11c-Cre - mice (right panels). (C) Representative FACS plots for granzyme B expression at d7 (upper panels) and d14 p.i. (lower panels) in Hif-1α flox/flox Cd11c-Cre + (left panels) and Hif-1α flox/flox Cd11c-Cre - mice (right panels). (TIF) [file ppat.1004938.s005.tif]

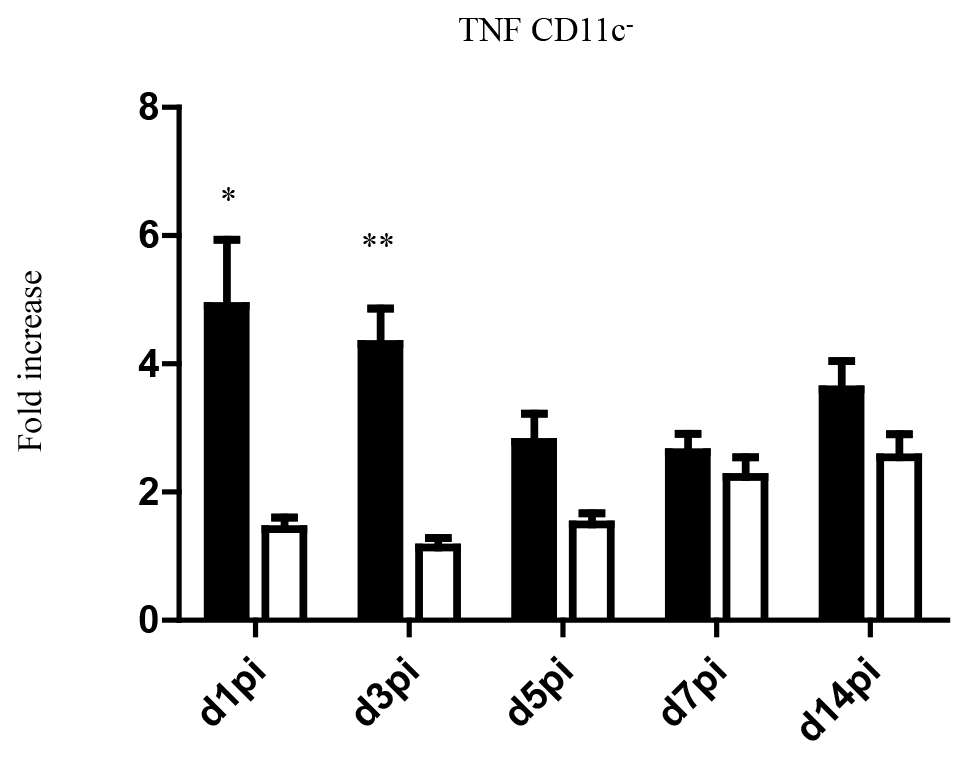

Supplement: S6 Fig — Real-time PCR analysis of I TNF expression in CD11c- cells from Hif-1α flox/flox Cd11c-Cre - and Hif-1α flox/flox Cd11c-Cre + mice over the course of infection. All data represent mean ± SEM combined from 3 independent experiments. (TIF) [file ppat.1004938.s006.tif]
